# Observations of supermicron-sized aerosols originating from biomass burning in southern Central Africa

**DOI:** 10.5194/acp-21-14815-2021

**Published:** 2021-10-06

**Authors:** Rose M. Miller, Greg M. McFarquhar, Robert M. Rauber, Joseph R. O’Brien, Siddhant Gupta, Michal Segal-Rozenhaimer, Amie N. Dobracki, Arthur J. Sedlacek, Sharon P. Burton, Steven G. Howell, Steffen Freitag, Caroline Dang

**Affiliations:** 1 Department of Atmospheric Science, University of Illinois Champaign-Urbana, Urbana, IL, USA; 2 Cooperative Institute of Mesoscale Meteorological Studies, University of Oklahoma, Norman, OK, USA; 3 School of Meteorology, University of Oklahoma, Norman, OK, USA; 4 Department of Atmospheric Science, University of North Dakota, Grand Forks, ND, USA; 5 Bay Area Environmental Research Institute/NASA Ames Research Center, Moffett Field, Mountain View, CA, USA; 6 Department of Geophysics, Porter School of Environmental and Earth Science, Tel Aviv University, Tel Aviv, Israel; 7 Department of Atmospheric Sciences, Rosenstiel School of Marine and Atmospheric Science, University of Miami, Miami, FL, USA; 8 Department of Environmental & Climate Sciences, Brookhaven National Laboratory, Upton, NY, USA; 9 Science Directorate, NASA Langley Research Center, Hampton, VA, USA; 10 Department of Oceanography, University of Hawai’i at Mānoa, Honolulu, HI, USA; 11 Universities Space Research Association/NASA Ames Research Center, Moffett Field, Mountain View, CA, USA

## Abstract

During the 3 years of the ObseRvations of Aerosols above CLouds and their intEractionS (ORACLES) campaign, the NASA Orion P-3 was equipped with a 2D stereo (2D-S) probe that imaged particles with maximum dimension (*D*) ranging from 10 < *D* < 1280 μm. The 2D-S recorded supermicron-sized aerosol particles (SAPs) outside of clouds within biomass burning plumes during flights over the southeastern Atlantic off Africa’s coast. Numerous SAPs with 10 < *D* < 1520 μm were observed in 2017 and 2018 at altitudes between 1230 and 4000 m, 1000 km from the coastline, mostly between 7–11° S. No SAPs were observed in 2016 as flights were conducted further south and further from the coastline. Number concentrations of refractory black carbon (rBC) measured by a single particle soot photometer ranged from 200 to 1200 cm^−3^ when SAPs were observed. Transmission electron microscopy images of submicron particulates, collected on Holey carbon grid filters, revealed particles with potassium salts, black carbon (BC), and organics. Energy-dispersive X-ray spectroscopy spectra also detected potassium, a tracer for biomass burning. These measurements provided evidence that the submicron particles originated from biomass burning. NOAA Hybrid Single-Particle Lagrangian Integrated Trajectory (HYSPLIT) 3 d back trajectories show a source in northern Angola for times when large SAPs were observed. Fire Information for Resource Management System (FIRMS) Moderate Resolution Imaging Spectroradiometer (MODIS) 6 active fire maps showed extensive biomass burning at these locations. Given the back trajectories, the high number concentrations of rBC, and the presence of elemental tracers indicative of biomass burning, it is hypothesized that the SAPs imaged by the 2D-S are examples of BC aerosol, ash, or unburned plant material.

## 1 Introduction

Global biomass burning (BB) emits on average 2.5 Pg yr^−1^ of carbon aerosol, with Africa producing 49% of these global emissions (van der Werf et al., 2006). Particulates generated by BB scatter and absorb solar radiation, affect the properties and lifetime of clouds (Andreae, 1991; Penner et al., 1992; Ackerman et al., 2000; Bond et al., 2013), and influence regional and global climate (Crutzen and Andreae, 1990; Andreae, 1991; Bond et al., 2013). Active fire detection from geostationary satellites over central Africa detect BB from woodland, cropland, and grassland fires, which typically peak in July with ~6 Tg d^−1^ of BB combustion (Roberts et al., 2009). Westward transport of aerosols from BB places the plume over an expansive seasonal stratocumulus cloud deck over the southeastern Atlantic Ocean (Muhlbauer et al., 2014). The products of BB, including soot aerosol, vary with vegetation type and emission during flaming or smoldering combustion. Previous studies on BB within central Africa observed an abundance of soot aerosols predominately from flaming grass fires (Li et al., 2003).

Black carbon (BC; e.g., soot) aerosol, a byproduct from incomplete combustion during biomass and fossil fuel burning (Bond et al., 2004), contributes to uncertainty estimates of radiative forcing in global climate models due to its optical property dependence on particle microphysical properties (Bond et al., 2013; Boucher et al., 2013). Combustion of biomass and fossil fuels can produce branching, chain-like (aciniform morphology) soot aggregates from submicron to supermicron sizes (Bond et al., 2004). Following particle generation, soot aggregates can undergo morphological changes that result in a collapse from a fractal structure to a more compact, spherical shape due to their interactions with H_2_O, H_2_SO_4_, and/or other gaseous species (Zhang et al., 2008; Shingler et al., 2016). Such physical modification will alter the optical properties of these particles (Martins et al., 1998; Reid and Hobbs, 1998; Weiss et al., 1992).

In addition, more KCl particles have been found in younger smoke aerosols, whereas more K_2_SO_4_ and KNO_3_ are present in older smoke aerosol samples. This process happens through reactions from other chemical species present in biomass burning (Li et al., 2003).

Large soot aggregates (> 1000 nm) have been observed in field studies of flaming wildfires over the southern Indian Ocean and the southwestern USA (Chakrabarty et al., 2014). Targeted laboratory experiments have yielded supermicron-sized soot aggregates ranging from 5 to 100 μm (Kearney and Pierce, 2012). This class of soot aggregates have also been observed in the emissions from the Kuwait oil fires in 1991 and were characterized by chain lengths up to 5 μm (Weiss et al., 1992). However, it is unknown whether these supermicron-sized aerosols can be transported long distances from their source regions.

In this study, we report observations of long-range transport of supermicron-sized aerosol particles (SAPs) originating from biomass burning in central Africa using measurements obtained aboard the NASA Orion P-3 research aircraft as part of the ObseRvations of Aerosols above CLouds and their intEractionS (ORACLES) field campaign (Redemann et al., 2021; Zuidema et al., 2016). Evidence from transmission electron microscopy (TEM) analysis of aerosol particles, BB aerosol composition analysis, particle shape and size, and prevailing atmospheric conditions together demonstrates that SAPs observed during this campaign are examples of supermicron-sized BC aerosol, ash, or unburned plant material.

## 2 Instrumentation/data

ORACLES missions were flown in 2016, 2017, and 2018. SAPs were not observed during the 2016 ORACLES field mission but were on 15 of 25 flights from 2017 and 2018. All of the 2017–2018 research flights were based out of the African island nation of São Tomé and Príncipe or Ascension Island in the South Atlantic, whereas the 2016 campaign was based out of Walvis Bay, Namibia.

The likely origin and transport path of the BB plume over the Atlantic Ocean were determined using the location of the observed SAPs during research flights along with the NOAA Hybrid Single-Particle Lagrangian Integrated Trajectory (HYSPLIT) backward trajectory model calculations. Analysis by Wu et al. (2020) suggests that the age of these African BB aerosols was greater than 7 d. A majority of the BB aerosols sampled during ORACLES were located in the free troposphere (Pistone et al., 2019). Remote sensing analysis of ORACLES has determined that there were no systematic differences in aerosol properties found between the air above low-level clouds and above nearby clear-sky areas during the daytime (Shinozuka et al., 2020).

### 2.1 ORACLES field campaign

ORACLES had three Intensive Observation Periods (IOPs) in successive years to study the atmospheric processes and climate impacts of African BB aerosols (Redemann et al., 2020). A seasonal BB plume is present between July and October, where BB aerosols are transported westward from Africa over the southeast Atlantic Ocean. The southeastern Atlantic hosts one of the three permanent subtropical stratocumulus cloud decks in the world. Understanding the impact and interactions between this aerosol plume and clouds has been emphasized in IPCC (Intergovernmental Panel on Climate Change) reports (Myhre et al., 2013). Aerosol–cloud interaction is one of the largest uncertainties in estimates of future climate from climate models. One of the objectives of ORACLES was to evaluate the interaction of these BB aerosols with stratocumulus clouds and determine their possible direct and indirect radiative effects.

The 2016 IOPs were based out of Walvis Bay, Namibia. No SAPs were detected during the 2016 IOPs. The 2017 and 2018 IOPs were based out of São Tomé. In these IOPs, 25 successful research flights were carried out in the region between 0–15° S and 12° E–15° W, which is further north than the 2016 flight domain. All SAPs discussed herein were detected within the BB plumes during these flights. Instrumentation to detect and measure aerosol properties was included in the payload of the P-3 aircraft for all 3 years.

### 2.2 2D-S stereo probe

Two-dimensional shadowgraph images with 10 μm pixel resolution were collected in situ from the 2D stereo (2D-S) optical array cloud probe (Lawson et al., 2006) about 800–1200 km west of Angola as part of all three ORACLES field campaigns. In total, 6 out of 12 research flights between 12 and 31 August 2017 detected SAPs within flight legs sampling biomass plumes above the stratocumulus cloud deck between 1230–4000 m above sea level. In 2018, 9 out of 13 research flights between 27 September and 23 October 2018 detected SAPs. Calibration of the instruments, which includes laser alignment and sizing, was performed by the manufacturer prior to and after each experiment. Instrument maintenance was performed within the field to ensure cleaned optics before each flight.

Particle images were processed using the University of Illinois/University of Oklahoma optical array processing software (UIOOPS; McFarquhar et al., 2018) and the Airborne Data Processing And Analysis (ADPAA; Delene et al., 2020) package (Delene, 2011). The 2D-S is normally used to capture cloud and ice particle images, but in this study, images corresponding to SAPs were collected in the biomass aerosol plumes. SAPs were identified with 2D-S imagery only during periods when the aircraft was not in cloud, which were indicated by periods when the King hot wire probe liquid water content was less than a zero offset value determined by analyzing data in cloud free air during each flight. The offset ranged from 0.031–0.036 gm^−3^. SAP images with a linear shape had canting angles (angle off of the direction of flight) of approximately 15–30° due to flow distortion around the probe head, which is a firm indicator that they have mass and were not artifacts. Canting is a well-known phenomena in 2D-S images. The 2D-S data were also examined when the aircraft was above the plume and in low concentrations of aerosol, and no false counts were found on the 2D-S, giving more confidence that the counts associated with the smaller SAPs are real. The absence of clouds in all areas where SAPs were observed was confirmed by examination of the forward video on the aircraft. For all SAPs, the longest dimension in any direction across the 3D volume of the particle (*D*) was recorded. *D* is equivalent to the diameter of the smallest sphere enclosing the particle (Wu and McFarquhar, 2016).

### 2.3 Cloud and aerosol spectrometer/cloud droplet probe

The cloud and aerosol spectrometer (CAS) measures smaller aerosol and cloud hydrometeor size distributions for 0.51 < *D* < 50 μm and relies on light-scattering rather than imaging techniques (Baumgardner et al., 2001). Data were processed using the Airborne Data Processing And Analysis (ADPAA) software package (Delene, 2011). In 2018, there was an instrument malfunction, and the CAS data were not usable for this research. However, data from a cloud droplet probe (CDP) are available for 2018. Like the CAS, the CDP measures size distributions from the forward scattering of light from target particles. It sizes particles between 2 and 50 μm.

### 2.4 Aerosol mass spectrometer

The Aerodyne time-of-flight aerosol mass spectrometer (ToF-AMS), operated by the Hawaii Group for Environmental Aerosol Research (HiGEAR), was used to determine non-refractory submicron aerosol composition within the BB plume by impaction of aerosols on a vaporizer. The AMS vaporizer temperature was set between 600 and 650 °C during individual flights to optimize evaporation of the organic aerosol (Howell et al., 2014; Shank et al., 2012; DeCarlo et al., 2006; Jayne et al., 2000). This was followed by electron ionization and time-of-flight mass spectral analysis. Size-resolved composition was quantified by measuring the arrival times of the aerosol at the vaporizer, as in Drewnick et al. (2005). ToF AMS data from the 2017 and 2018 campaigns were used to determine quantitative aerosol composition within the BB plume during the times that SAPs were observed by the 2D-S. The AMS inlet had a cutoff at approximately 700 nm. The applied collection efficiency, CE = max(0.5, 1−NH_4_/(2 × SO_4_)), neglected the small nitrate contribution, and used 0.5 as the lower limit, consistent with most field campaigns.

### 2.5 Single particle soot spectrometer

During the ORACLES campaigns a single particle soot spectrometer (SP2; Droplet Measurement Technologies; Revision D) was used to detect refractory black carbon (rBC) aerosol particles. The SP2 detects individual rBC particles through laser-induced incandescence (Schwarz et al., 2006; Moteki and Kondo, 2007). The incandescence signal can probe particles with mass equivalent diameter of rBC from nominally 80–500 nm, assuming a rBC density of 1.8 g cm^−3^. The SP2 was calibrated with fullerene soot (Alfa Aesar; stock no. 40971; lot no. F12S011). Soot is also commonly referred to as BC, elemental carbon (EC), light-absorbing carbon (LAC), and rBC (Buseck et al., 2012; Petzold et al., 2013). In the present paper, all these particle types are assumed to be equivalent, and the term soot is used to describe them, except for the BC noted on the aerosol filters.

### 2.6 P-3 aerosol inlet

The University of Hawaii’s shrouded diffuser inlet is thoroughly described in McNaughton et al. (2007) and was tested during the NASA DC-8 Inlet Characterization Experiment. The volumetric flow rate was proportional to airspeed, was maintained within 5% of the isokinetic flow rate, and had a shrouded constant area flow region around the inlet, with a 4.5° diffuser half-angle and a 3.8 cm inner diameter tube with the largest possible radius of curvature to complete a 45° bend to bring the air into the fuselage. McNaughton et al. (2007) found that the 50% transmission efficiency of the inlet is 3.1 μm geometric equivalent diameter (with a particle density of 2.6 g cm^−3^) at the surface and 2.0 μm at 12 km. These cutoffs were used for the SP2, AMS, and aerosol filter system (AFS).

### 2.7 Aerosol filter system

The AFS obtained aerosol filters which were then analyzed to provide the chemical composition of aerosols within the BB plume for the 2017 and 2018 campaigns. Particles collected on the filters were analyzed using transmission electron microscope (TEM) and energy dispersive x-ray spectroscopy (EDS) to determine morphology and composition following the techniques of past research for aerosols sized between 30 nm and 5 μm (Echalar et al., 1995; Gao et al., 2003). Filter samples were only collected within the BB plume. For 2017, only two filters were collected and analyzed with the above techniques as the other filters could not be analyzed because of technical problems.

### 2.8 NOAA HYSPLIT

NOAA’s HYSPLIT model, January 2017 revision (854) version (Draxler and Hess, 1998; Stein et al., 2015), was used to calculate air parcel backward trajectories to determine air mass source regions during the 2017 and 2018 campaigns. The HYSPLIT model was initialized with the Global Data Assimilation System (GDAS) at a 0.5° grid spacing. Backward trajectories were initiated at the altitude and location where the SAPs were observed; each backward trajectory was run until the air parcel was within 500m of the surface, during times which spanned between 48 and 128 h. The trajectories were used to determine the possible location of air parcels and establish source relationships between the BB plume and the observed SAPs. Since air parcels may be lofted through the boundary layer by the heat of fires, which is not accounted for in HYSPLIT, using 500m above the surface as the criteria for the back trajectory endpoints represents the easternmost locations where the parcels likely originated over Africa.

### 2.9 CALIPSO and HSRL

The Cloud-Aerosol Lidar and Infrared Pathfinder Satellite Observation (CALIPSO) satellite provided the location of clouds and atmospheric aerosol loading over the southeastern Atlantic region. CALIPSO combines an active lidar instrument with passive infrared and visible imagers to probe the vertical structure and properties of thin clouds and aerosols over the globe (Winker et al., 2003). For this research, CALIPSO data from an overpass on 30 August 2017 was used to show the presence of the low-level aerosol air mass being advected off the coast of central western Africa. On board the P-3 research aircraft for the 2017 and 2018 campaigns, there was a similar but more capable active lidar instrument, the High Spectral Resolution Lidar (HSRL-2), which can discriminate between aerosol and molecular signals to measure aerosol extinction, backscatter and depolarization, and clouds. The lidar instruments permitted characterization of the spatial and vertical distributions of the aerosols (Burton et al., 2018; Sawamura et al., 2017).

The HSRL-2 and Cloud-Aerosol Lidar with Orthogonal Polarization (CALIOP) lidar were used to identify the location of a large aerosol plume over the southeastern Atlantic Ocean. A large aerosol plume situated 1.5–4.0 km above the stratocumulus cloud deck was frequently observed both by the CALIOP in 2017 and HSRL-2 in both 2017 and 2018. In situ sampling of this aerosol plume was carried out by the P-3 research aircraft for 24 flights, with 15 flights measuring SAPs.

### 2.10 MODIS and FIRMS

Satellite remote sensing instrumentation was used to determine source origins for SAPs. The Fire Information of Resource Management System (NASA Firms, 2021) uses near real-time Moderate Resolution Imaging Spectroradiometer (MODIS) data to estimate thermal anomalies and fire locations. The MODIS collection 6 was processed by NASA’s Land, Atmosphere Near real-time Capability for Earth Observing System (EOS) using swath products (MOD14/MYD14). The thermal anomalies and active fires represent the center of a 1 km pixel that is flagged by the MODIS fire and thermal anomalies algorithm (Giglio et al., 2003) as containing one or more fires within the pixel.

## 3 Case studies

Case studies for two pairs of flights are presented, with one pair conducted on 30 and 31 August from the 2017 ORACLES campaign and a second pair conducted on 3 and 4 October from the 2018 ORACLES campaign. These flights were chosen as case studies because flights on the consecutive days provided the opportunity to sample the same plume and allow examination of how the BB plume located over the southeastern Atlantic Ocean evolved over time and whether this evolution influenced the way that the SAPs changed and aged.

### 3.1 30–31 August 2017

**Figure 1.**
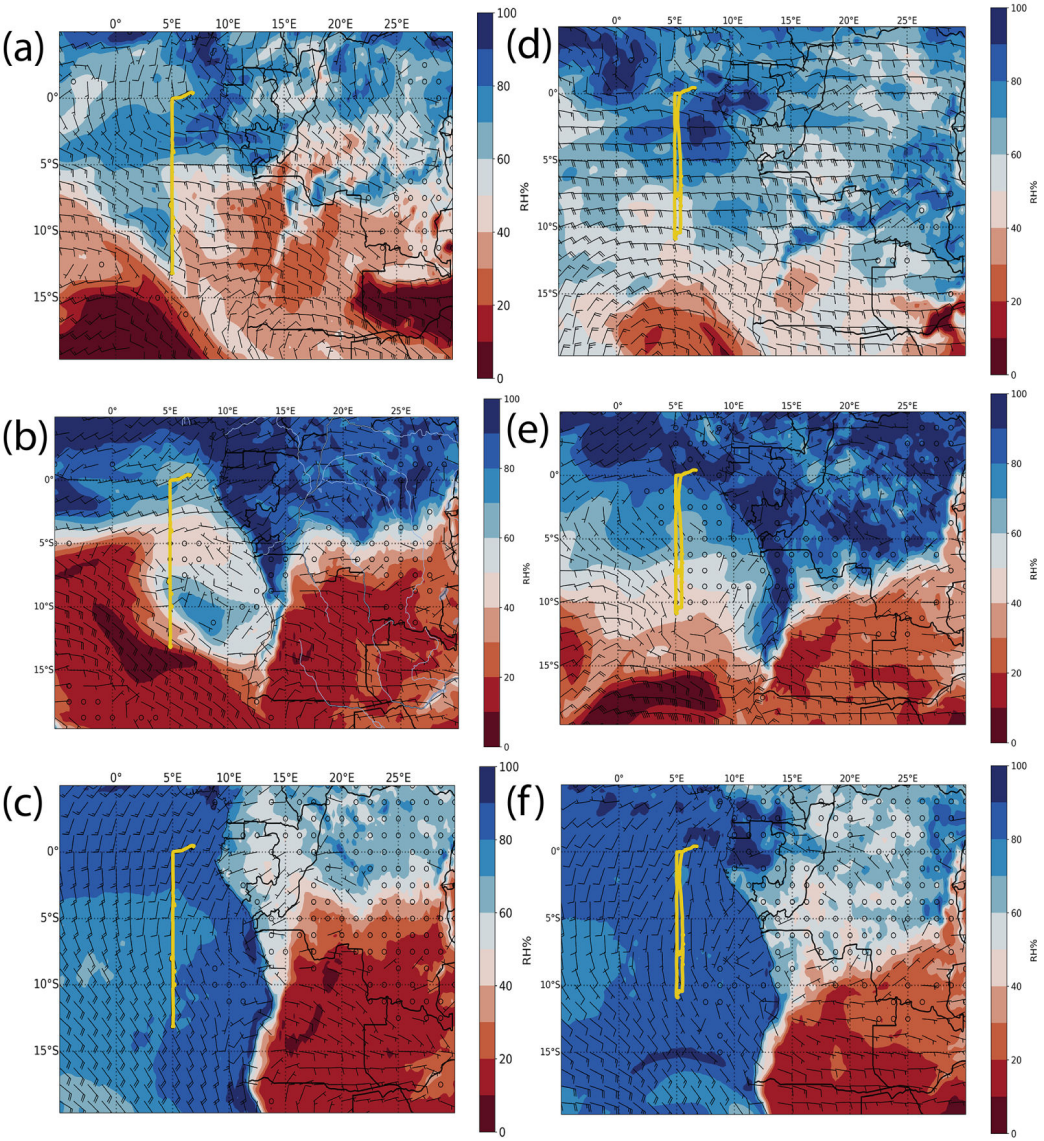
European Centre for Medium-Range Weather Forecasts 0 h reanalysis at 12:00 UTC on 30 August 2017 (**a–c**) and 2 October 2018 (**d–f**). (**a, d**) The 700 hPa relative humidity, along with wind. (**b, e**) The 850 hPa relative humidity and wind. (**c, f**) Surface relative humidity and wind. Wind barbs are as follows: small barbs are 5ms^−1^, and large barbs are 10ms^−1^. The flight track is shown as a yellow line.

**Figure 2.**
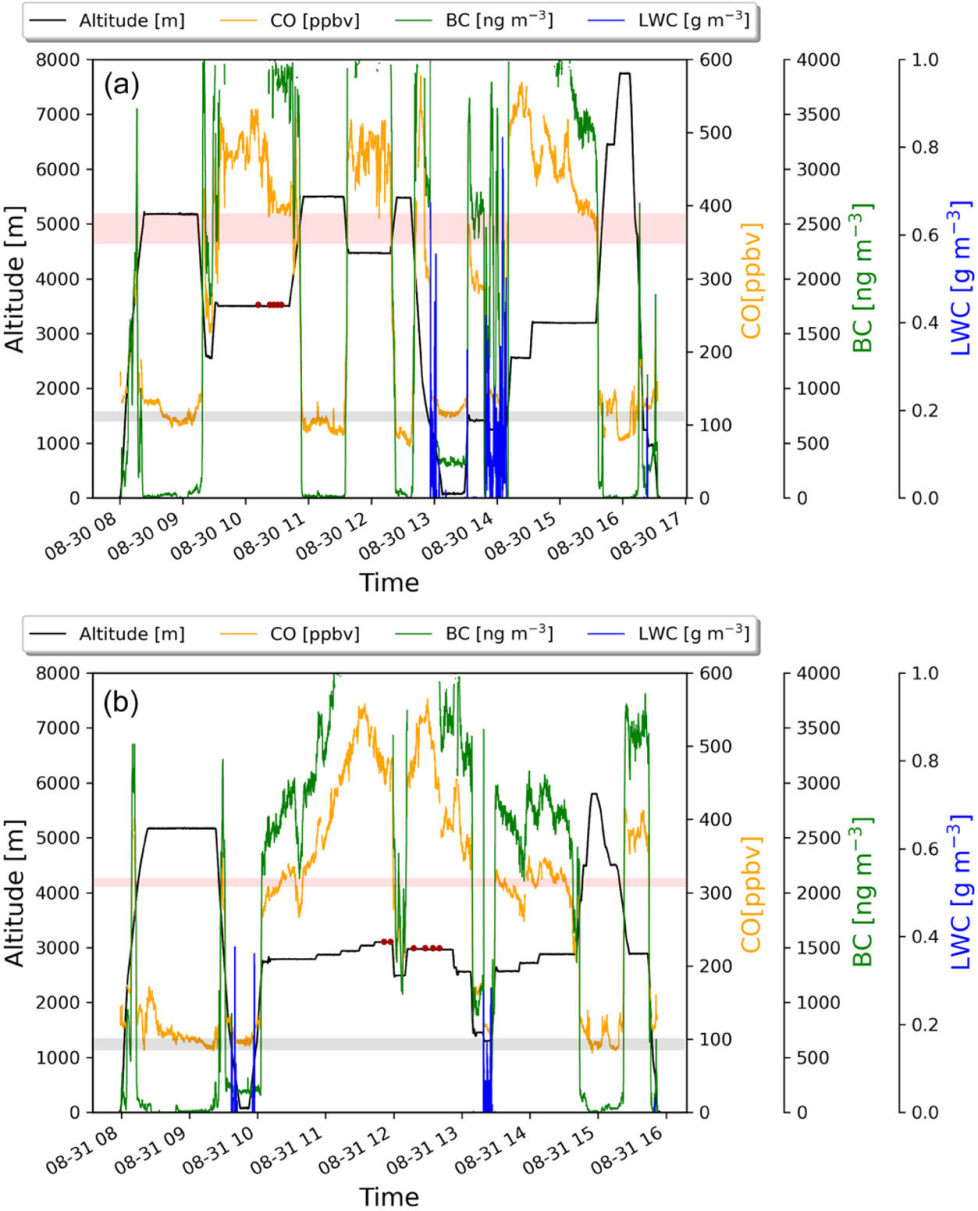
(**a**) Time series of RF 11, together with the aircraft altitude (black line), carbon monoxide (yellow line), black carbon (green line), cloud liquid water content (blue line), height range of the top of the biomass burning plume (pink bar), height range of the top of the stratocumulus deck (gray bar), and locations where SAPs were observed (red dots). (**b**) Same as (**a**) but for RF 12.

The two sequential research flights, RF 11 and RF 12, occurred on 30 and 31 August. In Fig. 1a-c, 700 hPa (~3000 m), 850 hPa (~1500 m), and surface maps for 30 August at 12:00 UTC are shown. The wind fields in Fig. 1a-c together show that the BB plume was only transported westward over the stratocumulus deck at higher levels after the central plume was lofted to around 3000 m. The time series of carbon monoxide (CO), BC, and cloud liquid water content (LWC) for RF 11 and RF 12 (Fig. 2) shows that the SAP observations were clustered and found in a region well within the BB plume and above the cloud, where CO concentrations ranged from 375 to 500 ppbv and BC concentrations ranged from 3500 to 3750 ngm^−3^.

**Figure 3.**
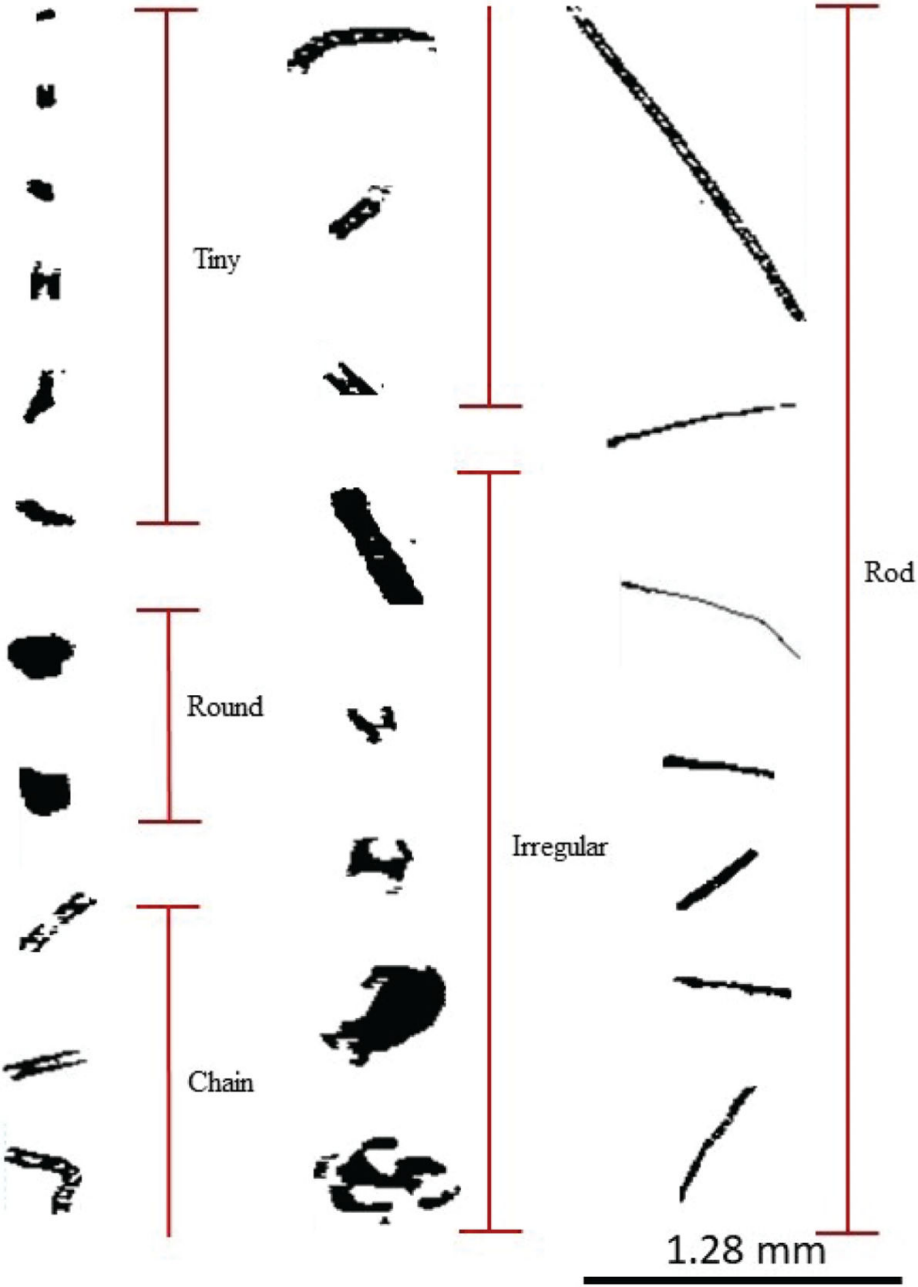
SAPs recorded within the BB plume by the 2D-S probe during the ORACLES 2017 campaign. SAPs were observed to be a variety of sizes and shapes, ranging from 10 to 1280 μm in length. The figure shows the five primary shapes of SAPs observed during 30–31 August 2017.

**Figure 4.**
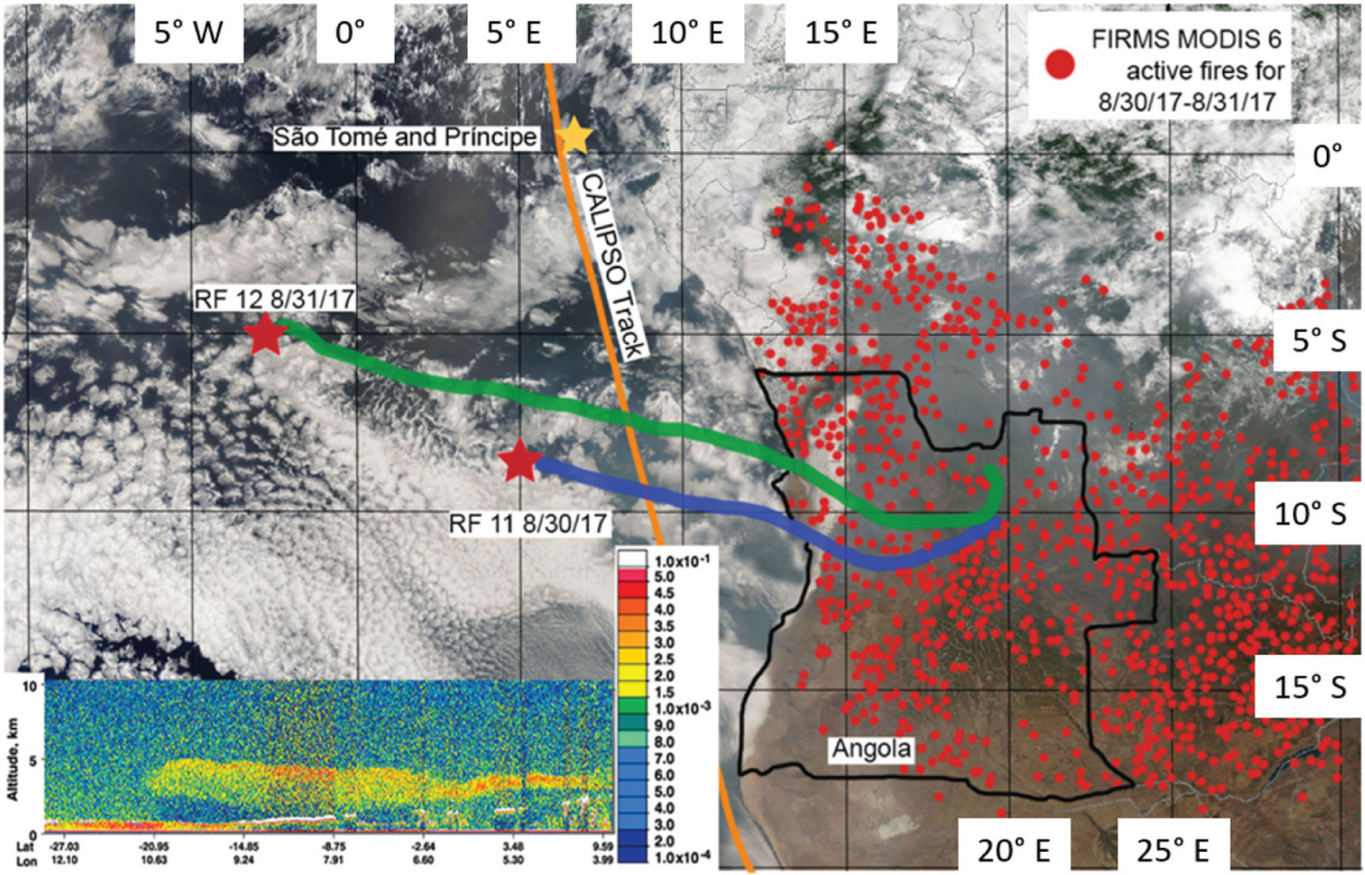
NOAA HYSPLIT backward trajectories from RF 11 and RF 12 overlain with visible imagery of south central Africa from the Moderate Resolution Imaging Spectroradiometer (MODIS) sensor aboard the Terra satellite at 09:40 UTC for 30–31 August 2017 (source: image obtained from NASA near real-time (NRT) data archive). Fire Information for Resource Management System (FIRMS) MODIS 6 active fire hot spots for 30–31 August 2017 (red dots) are shown. The inset shows the 532 nm attenuated backscatter return signal from the Cloud-Aerosol Lidar with Orthogonal Polarization (CALIOP) lidar aboard the Cloud-Aerosol Lidar and Infrared Pathfinder Satellite Observation (CALIPSO) satellite, which shows the vertical distribution of aerosols on 30 August 2017 (source: image obtained from NASA CALIPSO data archive). The color scale indicates the strength of the lidar return signal, 532 nm total attenuated backscatter, in kilometers steradian (hereafter km^−1^ sr^−1^), where the boundary layer clouds tops are white and aerosols are green, yellow, and red.

In RF 11, 71 SAPs were measured that ranged in size between 10 and 250 μm along their largest dimension *D*. Each SAP was categorized based on its size and shape, (i.e., tiny, round, chain, rod, or irregular; Fig. 3). These SAPs were mostly tiny and irregular in shape. During RF 11, the plume was sampled at 3500m altitude at 5° E, and between 4–10° S. In contrast, in RF 12, about 24 h later, 12 SAPs with *D* between 800 and 1520 μm were detected at 2500m at 2.2° W, and 5° S, further west of Angola, as denoted by the red stars in Fig. 4. These larger SAPs were rods and chains.

**Figure 5.**
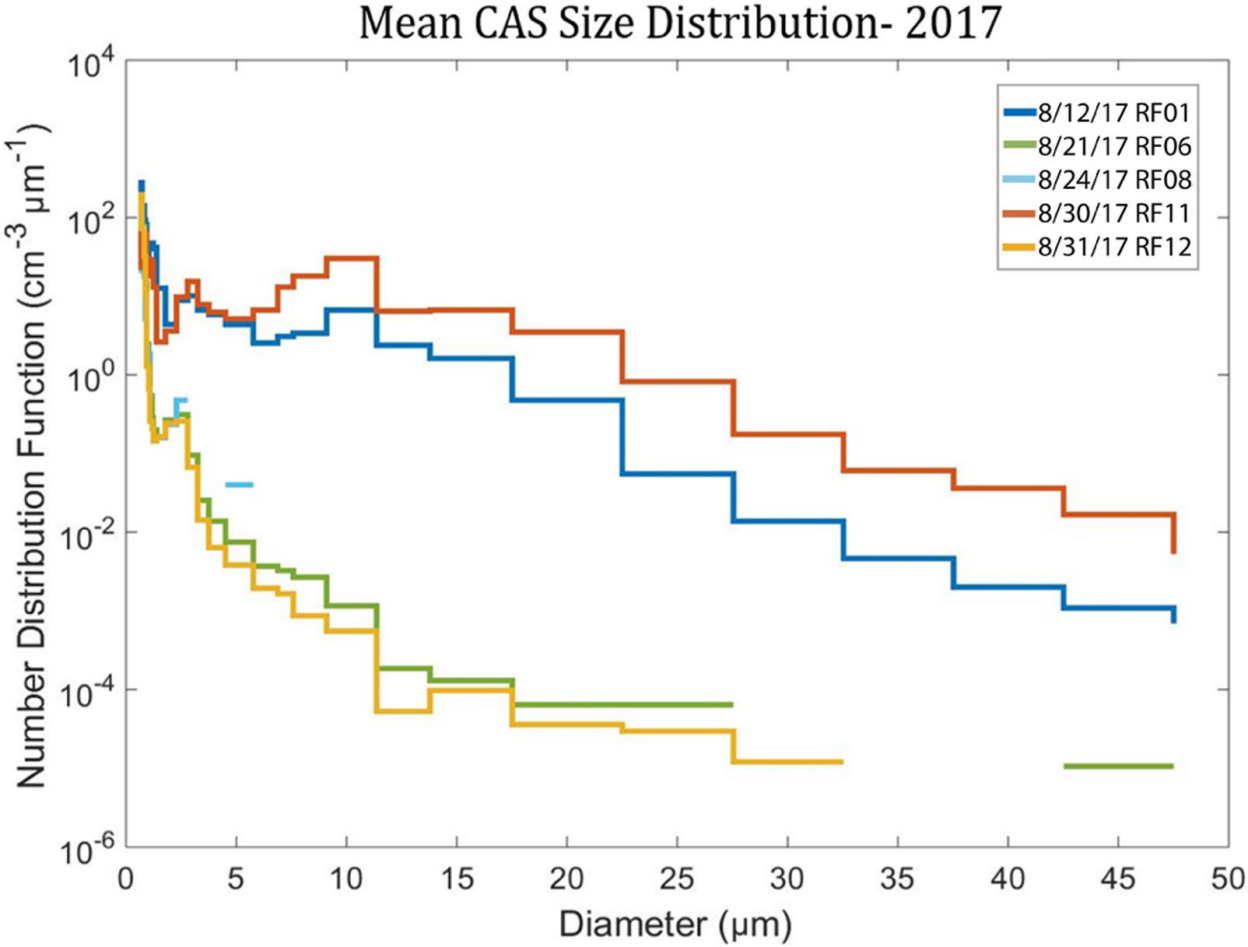
Mean cloud and aerosol spectrometer (CAS) aerosol size distribution for five research flights during a 5 min interval when 2D-S SAPs were measured within the BB plume.

During the flights the biomass burning plume layers were identified from HSRL imagery. Selected layers were then sampled by in situ probes as the P-3 flew through the plume. At each time that SAPs were detected with the 2D-S imagery on these flights, the cloud and aerosol spectrometer (CAS) observed aerosols with *D* ranging from 0.51 to 50 μm. The CAS size distributions in Fig. 5 during these time periods represent aerosol distributions outside of the cloud, as confirmed by examination of the forward video camera. During RF 11, about 100 cm^−3^ more particles were measured in the larger bin sizes between 10–50 μm compared to RF 12. The CAS operates as a forward-scattering probe, while the 2D-S imaging is based on occultation of a photo diode array based on 50% occultation. The fact that the CAS is seeing higher concentrations strongly suggests that the 2DS is missing many of the smaller particles near 10–20 μm because they are not sufficiently occulting the photo diode array. The CAS did not report any counts in clear air above the aerosol plume.

**Figure 6.**
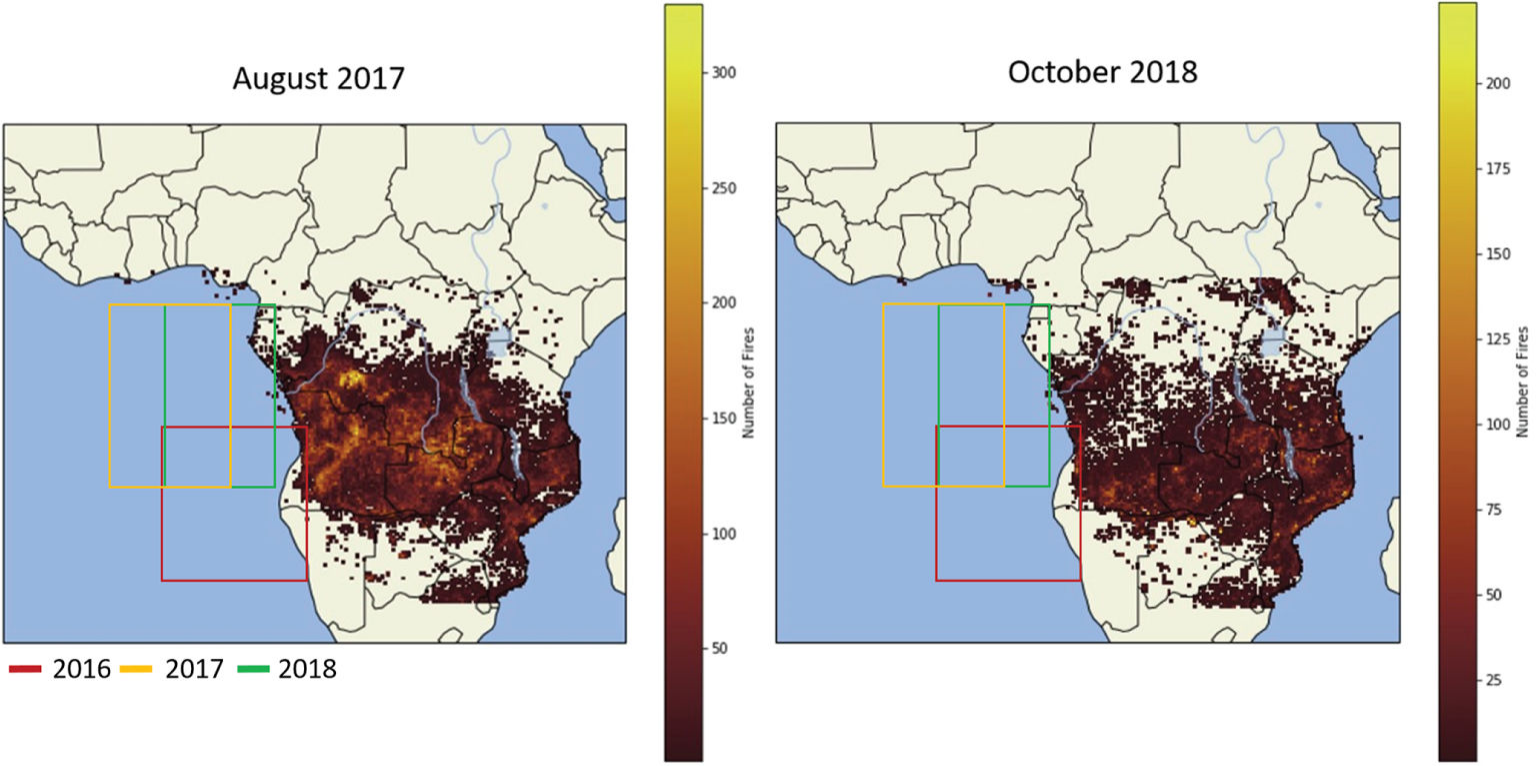
FIRMS MODIS 6 active fire map data for the months of August 2017 and October 2018, showing extensive biomass burning in central Africa. The boxes show the 2016 flight area (red), the 2017 flight area (yellow), and the 2018 flight area (green).

From the location of the SAPs, HYSPLIT 48 h backward trajectories were calculated to estimate the origin of the air parcels containing the SAPs. Trajectories from both locations showed that SAPs measured at an altitude of 3500m (RF 11) and 2500m (RF 12) had their respective air parcels passing over northern Angola about 2–3 d earlier, respectively (Fig. 4). The recorded FIRMS active fires from 30 to 31 August shows a large number of active fires throughout Angola and central Africa and, therefore, a large source of biomass smoke entering the atmosphere (Fig. 6). On 30 August, a CALIPSO overpass captured a large aerosol plume between 1.5 and 4.0 km above sea level transported westward over the Atlantic Ocean and over the stratocumulus cloud deck from the many fires from Angola and central Africa (Fig. 4). Note that, on Fig. 6, the southern two-thirds of the 2016 flight area was west of the Namib desert, and the northern third was west of the southernmost part of the BB region in Africa. Because the 2016 flights departed from Walvis Bay, the residence time of the flight in the BB plume was short compared to the 2017 and 2018 flights. This may be the reason that no SAPs were observed in 2016.

The altitude and location within the BB plume that the SAPs were observed for RF 12 occurred at 2500m and 820 km west of Angola. Larger diameter SAPs (*D* > 50 μm) were observed on the 2D-S during the RF 12 flight. These would have not been detected by the CAS. The particles found on RF 12 most likely were either supermicron-sized BC aerosol, ash, or unburned plant material formed near the fire and transported in the BB plume over the Atlantic Ocean.

**Figure 7.**
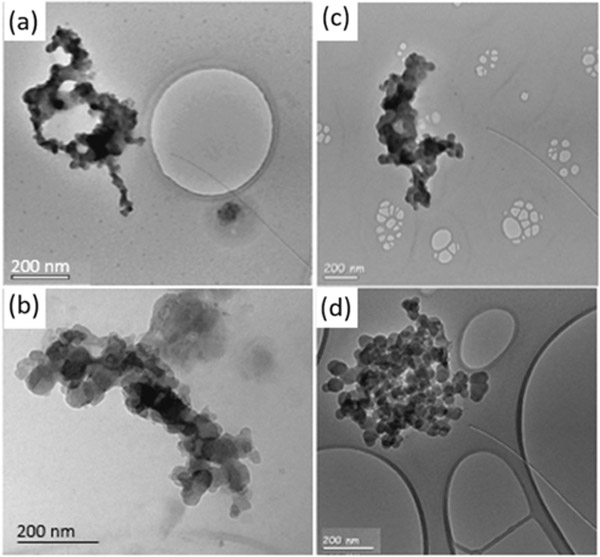
Transmission electron microscope/microscopy (TEM) images of black carbon particle aggregates collected on 30 August 2017 (**a**), 31 August 2017 (**b**), 2 October 2018 (**c**), and 3 October 2018 (**d**) on copper grid polycarbonate filters.

**Figure 8.**
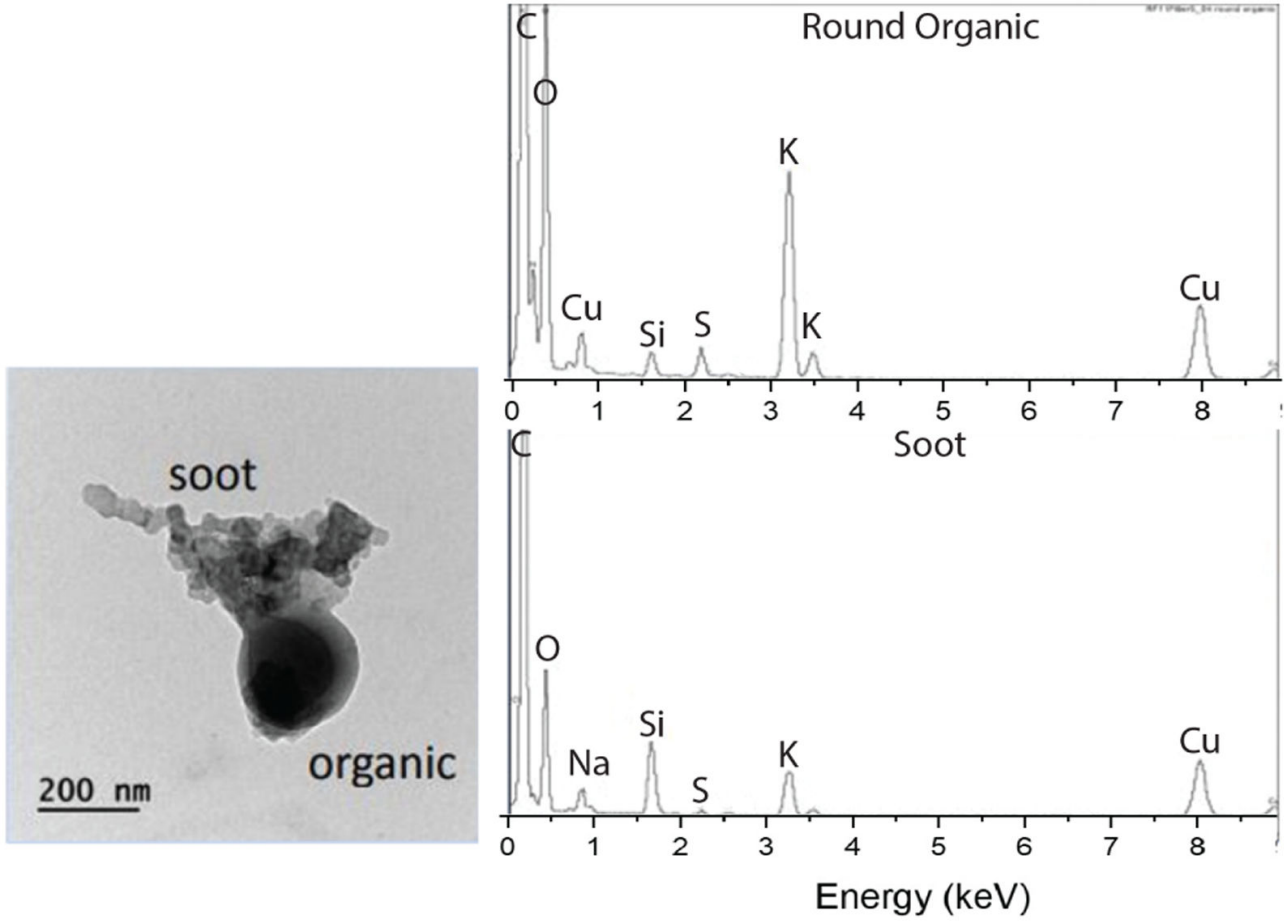
Energy-dispersive X-ray spectroscopy (EDS) elemental analysis of an organic/soot particle from RF 11 on 30 August 2017.

Filter samples were acquired by the AFS during the same time that the 2D-S observed SAPs during RF 11 and RF 12 (Fig. 7). The filter samples from both RF 11 and RF 12 contained commonly observed accumulation-mode soot aggregates, organic matter, and dust, all with *D* < 3.1 μm because of the inlet cutoff, significantly smaller than the particles measured by the 2D-S. Energy-dispersive X-ray spectroscopy (EDS) was performed on aerosol deposited on the filters (Fig. 8). The soot particles showed carbon, sulfur, oxygen, and silicon inclusions and the organic particles contained potassium, which are characteristic of biomass burning emissions (Pósfai et al., 2003). The filters contained numerous BC and organic particles that were captured during the time that the 2D-S observed SAPs. It is, therefore, likely that the SAPs are examples of supermicron-sized BC aerosol, ash, or unburned plant material.

### 3.2 2–3 October 2018

**Figure 9.**
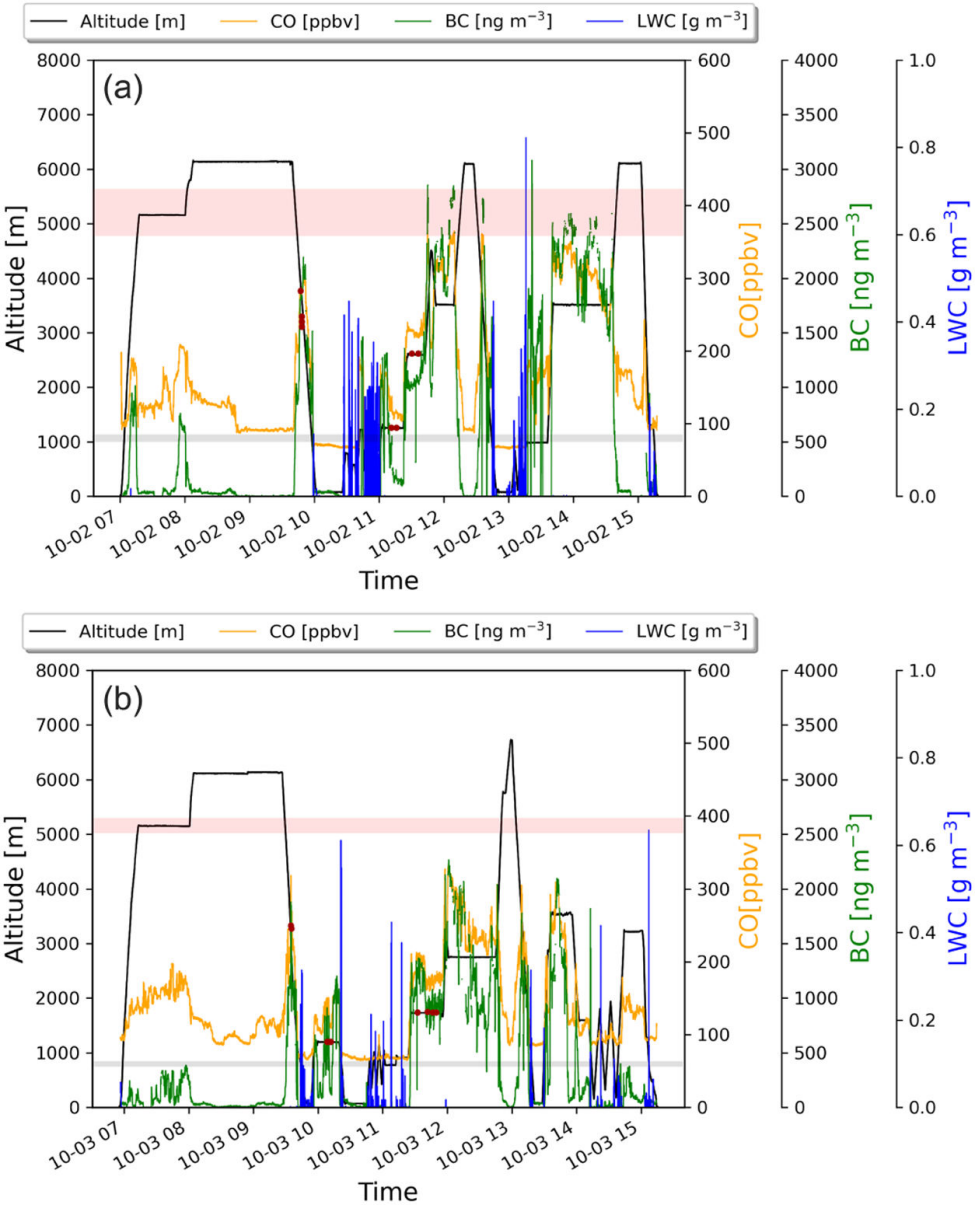
(**a**) Time series of RF 03, with the aircraft altitude (black line), carbon monoxide (yellow line), black carbon (green line), cloud liquid water content (blue line), height range of top of BB plume (pink bar), height range of top of stratocumulus deck (gray bar), and locations where SAPs were observed red (dots). (**b**) Same as (**a**) but for RF 04.

The RF 03 and RF 04 case studies presented here occurred on 2 and 3 October 2018 and, like the 2017 case studies, were two sequential flights where SAPs were observed. The wind fields at the surface, 850, and 700 hPa were similar to 2017 in that the BB plume was only transported westward over the stratocumulus deck at the higher altitude (Fig. 1d-f). The time series of CO, BC, and cloud LWC for RF 03 and RF 04 (Fig. 9) shows that the SAP observations were again clustered but at three different altitudes. In RF 11, one cluster, observed at 4000 m, had CO concentrations near 300 ppbv (parts per billion by volume) and BC concentrations near 3000 ngm^−3^. The second cluster, observed at 2800 m, had CO concentrations near 225 ppbv and BC concentrations near 1000 ngm^−3^. The third cluster, observed about 100m above cloud top, had CO concentrations near 110 ppbv and BC concentrations near 475 ngm^−3^. In RF 12, a cluster at 3500m had CO concentrations near 275 ppbv and BC concentrations near 2000 ngm^−3^. The second cluster at 1750m had CO concentrations near 175 ppbv and BC concentrations near 1000 ngm^−3^. The third cluster at 1200m had CO concentrations near 150 ppbv and BC concentrations near 800 ngm^−3^. All SAP observations were within the BB plume and above the cloud, with higher altitude clusters more centralized in the plume based on the BC and CO concentrations.

**Figure 10.**
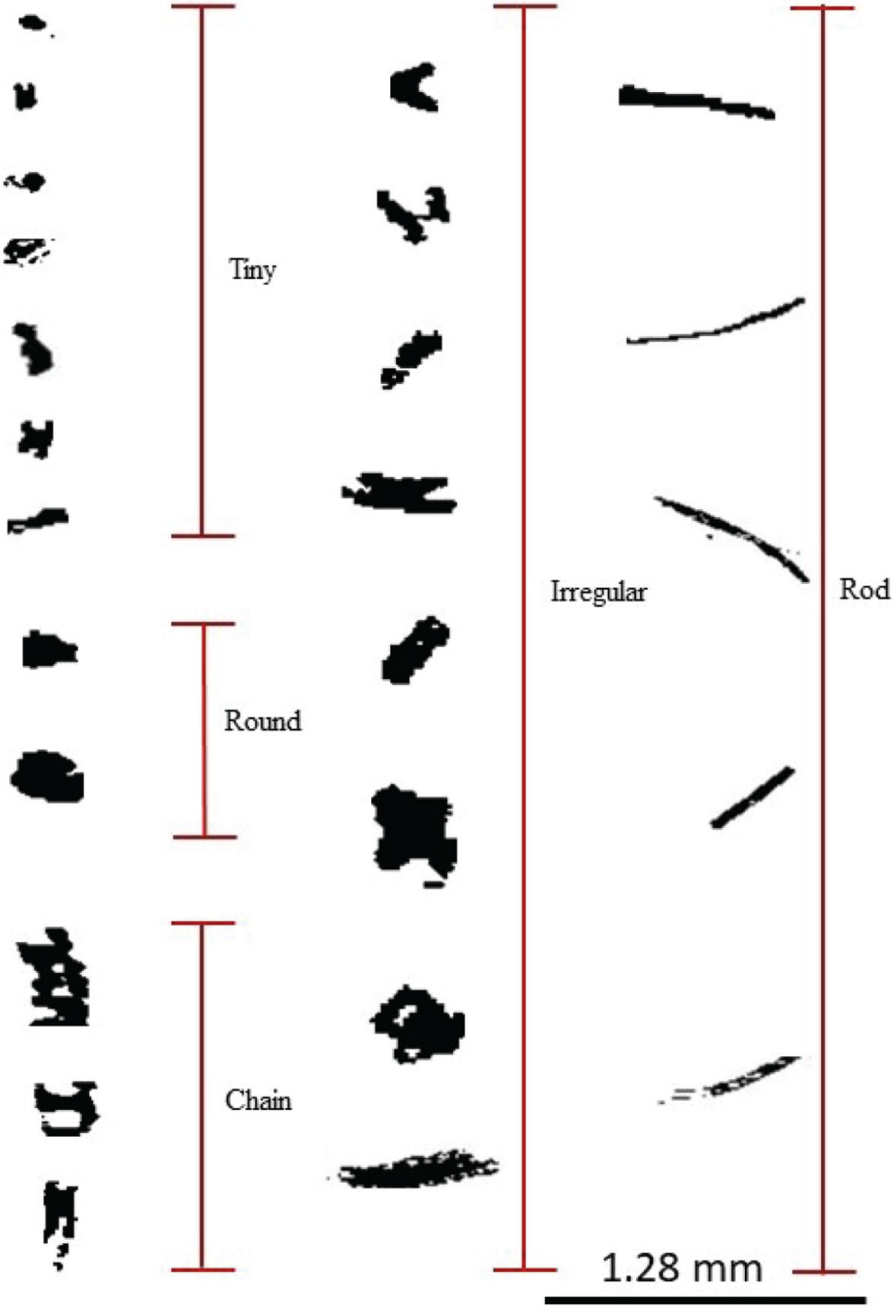
SAPs recorded within the BB plume by the 2D-S probe during the ORACLES 2018 campaign. SAPs were observed to be a variety of size and shapes, ranging from 10–1000 μm in length. The figure shows the five primary shapes of SAPs observed during 2–3 October 2018.

RF 03 sampled 102 SAPs ranging from 10 < *D* < 350 μm, mostly tiny, irregular, and round, and three large chain and irregular SAPs (700–1000 μm), while RF 4 sampled 48 SAPs with 600 < *D* < 1000 μm, which were mostly chain and rod SAPs (Fig. 10).

**Figure 11.**
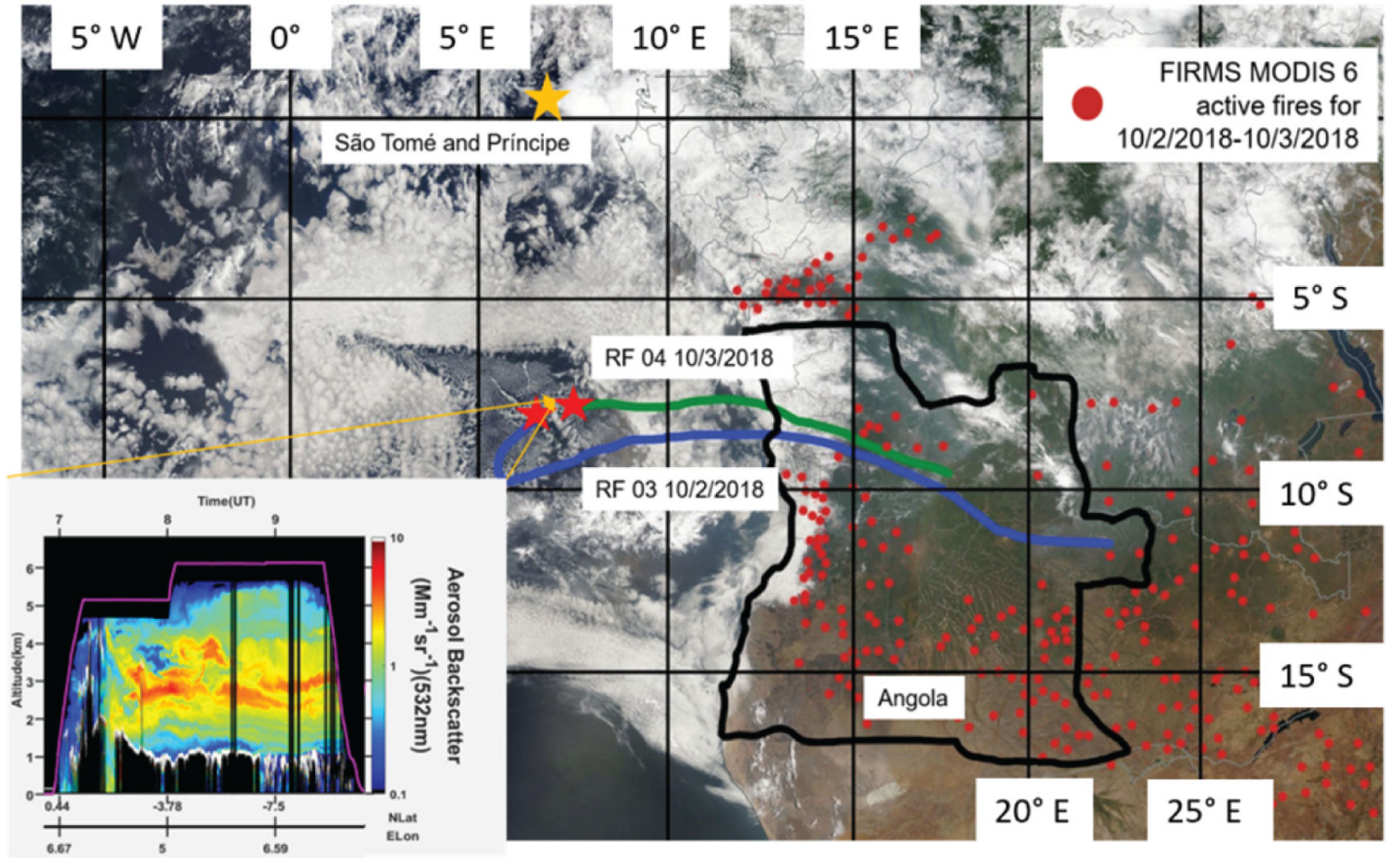
Ensemble-averaged NOAA HYSPLIT backward trajectories from RF 03 and RF 04 overlain with visible imagery of southern central Africa from the MODIS sensor aboard the Terra satellite for 2–3 October 2018 (source: image obtained from NASA NRT data archive). FIRMS MODIS 6 active fire hot spots for 2–3 October 2018 (red dots) are shown. The inset shows 532 nm backscatter return signal from the HSRL lidar aboard the P-3 research aircraft, showing the vertical distribution of aerosols on 3 October 2018. The color scale indicates the aerosol backscattering coefficient, with boundary layer cloud tops as white and aerosols as green, yellow, and red (indicating low, medium, and high loads, respectively).

The FIRMS MODIS 6 active fire map data for 2 and 3 October 2018 showed fewer active fires compared to 2017, which were all shifted further south due to the start of the rainy season in central Africa (Fig. 6b). RF 03 and RF 04 in 2018 occurred in a similar sampling region (centered at 5° E and 7° S) as opposed to RF 11 and RF 12 during 2017, which sampled different areas. Backward air parcel trajectories from the location of the SAPs (red stars) placed the source region in central Angola for RF 04 and eastern Angola for RF 03 (Fig. 11). The production of SAPs may be related to fire intensity as very intense fires can inject more material into the atmosphere, as seen in SAFARI-92 (Le Canut et al., 1996). The SAPs observed in RF 03 had smaller *D* overall compared to RF 04, which recorded the largest particle length of 1000 μm. The larger particles of RF 04 were also measured at an altitude of 1300 m, about 1200m lower in elevation than the particles observed in RF 03 at 2500 m. Larger particles were more often sampled at the bottom layer of the BB plumes due to gravitational settling.

## 4 Discussion of 2017–2018 observations

**Figure 12.**
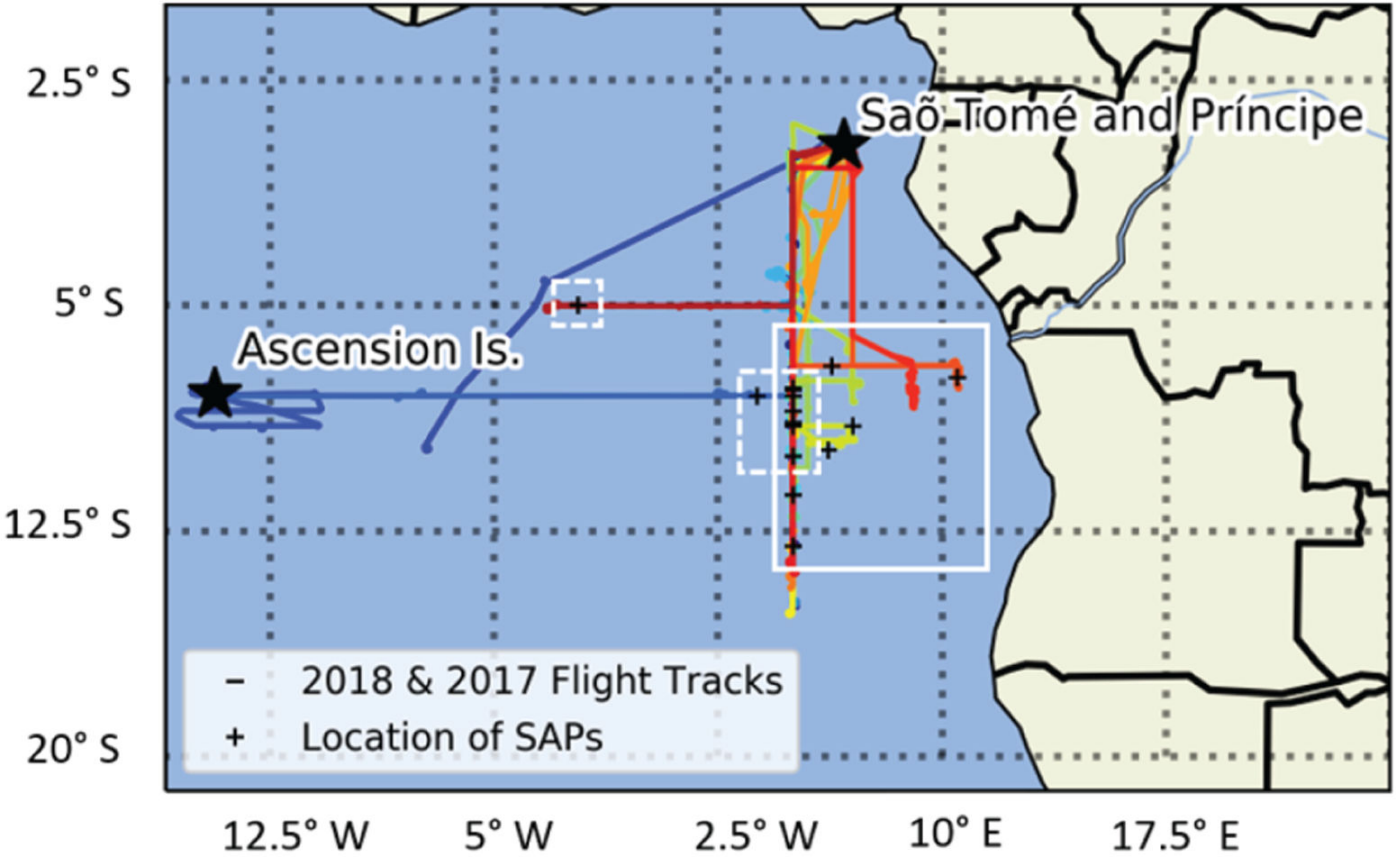
Research fight tracks from the 2017 and 2018 Intensive Observation Periods (IOPs) of ORACLES based out of São Tomé and Príncipe over the southeastern Atlantic Ocean (colors show flight tracks on different days). The 2017 and 2018 IOPs were off the coast of Angola. A few flights were conducted westward to Ascension Island for clear air sampling. Locations of observations of SAPs are denoted by the plus signs (+). The areas of observed SAPs in 2017 are denoted by the dashed white line and in 2018 by the solid white line.

The locations and conditions where SAPs were measured varied widely during flights but, when observed, were always observed within the BB aerosol plume throughout the 2017 and 2018 campaigns (Fig. 12). In 2017, the sampling area was between 5–9.5° S and 14° W–5° E. Research flights conducted in 2018 covered a larger area compared to 2017, with a latitude and longitude range from 1–13° S and 5–10.5° E, sampling about 420 km closer to the coast of Angola. The altitude of the in-plume sampling ranged between 1230–4000m for 2017, with SAPs measured lower in the plume between 1230–3500 m. The 2018 plume sampling ranged between 1300–2500 m, with SAPs measured throughout that range. The differences in locations sampled between 2017 and 2018 may have impacted the number and size of SAPs recorded and the chemical composition of the overall BB plume.

The 2017 flights took place in August as the biomass burning season ramped up within central Africa. The 2018 flights were conducted in the month of October, which is the end of the biomass burning season. In 2018, SAPs were observed along the same 5° E longitudinal line as during 2017 but were found over a larger latitudinal range compared to 2017, namely between 3 and 15° S, compared to between 8 and 9° S for 2017. In 2018, flights were conducted closer to the coast of Angola than in 2017, but no SAPs were detected by the 2D-S probe closer to the coast; they were only observed further west around 5° E (Fig. 12). The sampling was conducted at altitudes lower than 2000m closer to the coast. From Fig. 1, the winds at these lower levels were less likely to transport the BB plume to regions sampled by the aircraft. During 2018, the fires in central Africa were less numerous as this was near the end of the BB season in the region. More SAPs were measured overall in 2018 than 2017.

Tables 1 and 2 summarize the data and the range of altitudes, temperatures, and locations at which the SAPs were measured, along with the number of SAPs, the chemical composition of the plume in which they were embedded, the HYSPLIT source areas, and the plume age estimated from HYSPLIT trajectories and WRF model runs described in Mallet et al. (2019). The chemical composition of the aerosol plume within an averaged 5 min time span varied widely on days where SAPs were or were not observed, as seen in Tables 1 and 2. For example, in Table 1, days that did not record SAPs had concentrations of organics between 2.1 and 25 μgm^−3^ compared to days where SAPs were measured that had concentrations from 18 t 50 μgm^−3^. Aerosol plumes where SAPs were observed had an overall larger concentration spread of organics, ammonium, sulfates, nitrates, and soot (Table 1).

**Figure 13.**
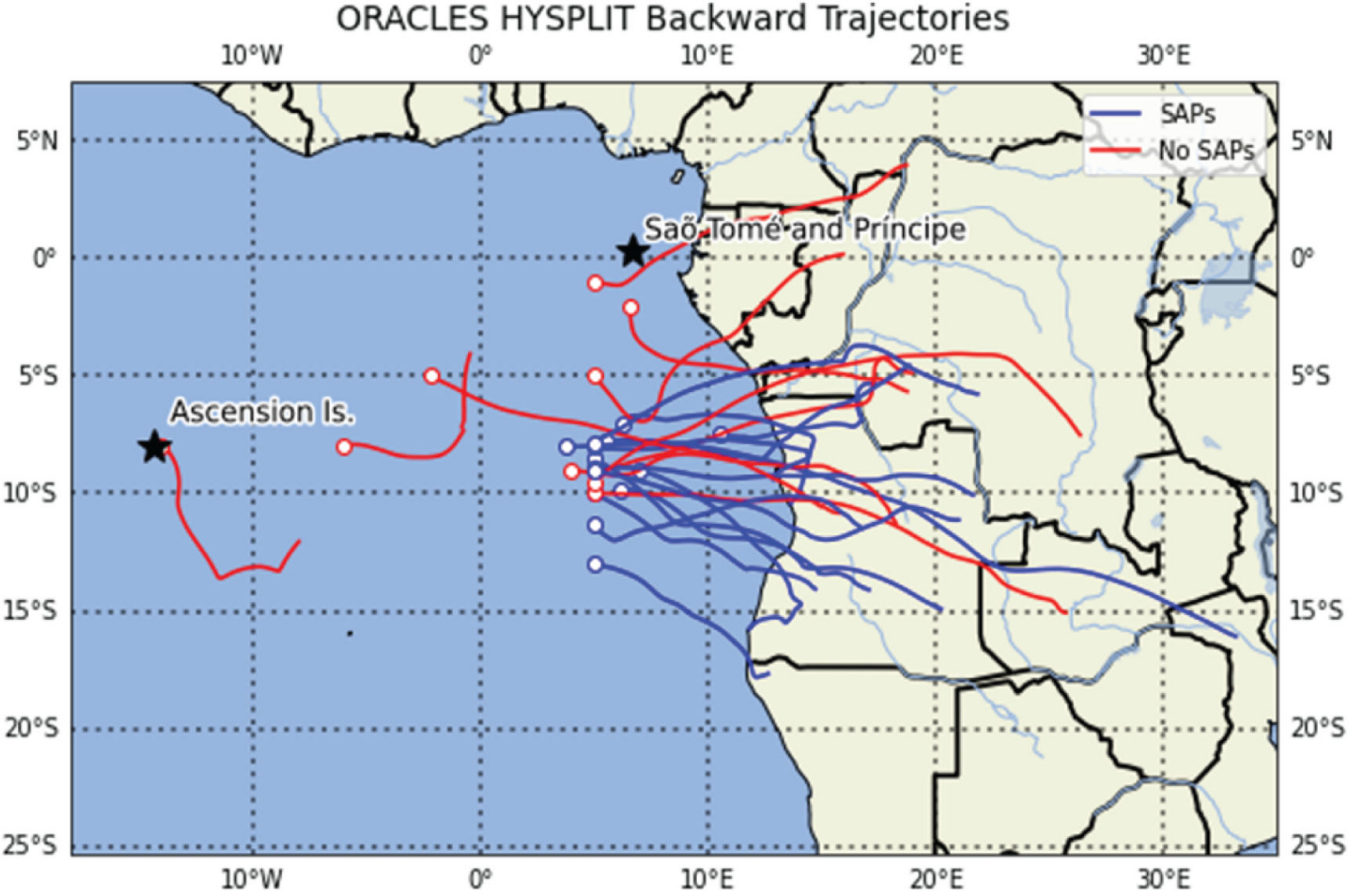
Location of 2017 and 2018 aerosol sampling legs and their 48 h HYSPLIT backward trajectories. Backward trajectories of SAPs (blue) show a possible source air mass region in northern and central Angola.

Plume age was estimated from the backward trajectories run with HYSPLIT and ranged from 2 to 6 d (Tables 1 and 2). From HYSPLIT, backward trajectories were run until the air parcel was within 500m of the surface. In 2018, the number of SAPs observed in relation to plume ranged widely. For example, 205 SAPs were measured on 27 September 2018 within a plume estimated to be 4.5 d old, whereas 102 SAPs were measured on 2 October 2018 within a plume estimated to be 2 d old. The complete set of backward trajectories from all of the research flights conducted showed Angola was the most common source region in both 2017 and 2018 (Fig. 13).

**Figure 14.**
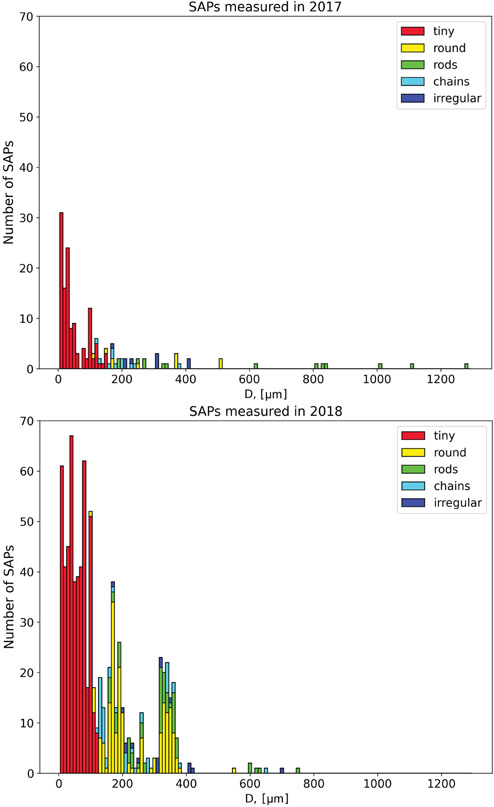
Particle sizes and shapes for the 165 SAPs measured in 2017 and the 692 SAPs measured in 2018.

The SAPs observed during 2017 and 2018 had maximum dimensions between 10 and 1280 μm. In 2017, 165 SAPs were measured on 6 of 12 research flights, while in 2018, 692 SAPs were measured on 7 of the 13 research flights (Tables 1 and 2). Analysis of the SAP sizes measured each year illustrate that the SAPs most commonly had *D* < 200 μm (Fig. 14). These were too small to determine a definitive shape and were classified as tiny. The larger SAPs had chain-like or rod-like shapes. From 2017, five of the six research flights containing SAPs also contained aerosols observed with the CAS (Fig. 5). On flights within BB plumes, where SAPs were not observed by the 2D-S, the CAS instrument also did not detect aerosols, which implies that aerosols within plumes were sized < 0.51 μm.

In 2017, organic concentrations trended lower for the days that did not record SAPs. Concentrations were measured between 2.1 and 25 μgm^−3^ on days without SAPS, compared to 18–50 μgm^−3^ on days with SAPs (Table 1). In addition, nitrates, sulfates, and ammonium concentrations were also lower for days with no recorded SAPs. This suggests that BB plumes with higher concentrations of organics, nitrates, sulfates, and ammonium were more likely to contain SAPs as well.

In 2017, plume age was a major indicator of the presence of SAPs. Plumes aged between 2–3 d were the most likely to contain measurable SAPs. Flights flown through BB plumes aged between 3–8 d did not result in any SAPs observed on the 2D-S (Table 1). This suggests that either the SAPs were removed by gravitational settling or were not produced due to a difference in fuel type, fire intensity, or wind speed and direction from the fire origin.

The maximum height of the aerosol plumes measured by CALIOP over southern central Africa during the months of August and October was, on average, 5500m (Das et al., 2017). Assuming the SAPs were lofted to the top of the plume, their range of fall speeds can be estimated with the following bounds. The range of fall distance was estimated as being the distance between the top of the plume and the top and bottom P-3 altitudes where SAPs were observed. The lowest altitude that SAPs were observed was 1230 m, and the highest was 3500 m. The bounds for the fall speed times were the longest and shortest times from the HYSPLIT back trajectories, which ranged from 48 to 144 h. The range of fall velocities were then calculated to be, at most, between 24.7 and 0.4 cm s^−1^.

In 2018, higher concentrations of organics (1.2– 20 μgm^−3^) and nitrate, sulfate, and ammonium corresponded to times with measurable SAPs (Table 2). Out of the 10 research flights that recorded chemical composition consistent with BB, only two produced no observable SAPs on the 2D-S. There were too few days with SAPs during the 2018 campaign to determine whether chemical composition, plume age, or temperature was related to the presence of SAPs in the BB plume. The aerosol filter system provides the best insight concerning the composition of the BB plume and individual particles. The filter chemical analysis from 2017 shows that the chemical composition of the particles was consistent with BB burning, specifically as the EDS analysis showed peaks of K and Si, both indicators of BB. In addition to the filter evidence, the HYSPLIT back trajectories showed a source location over areas of BB.

The main difference between 2017 and 2018 was the number of active fires present in southern central Africa at the time of the flights. Figure 5 illustrates the number of active fires present during the months of August 2017 and October 2018. In 2017, the entire southern central region of Africa around Angola, the Democratic Republic of Congo (DRC), and Zambia had between 200 and 300 active fires of unknown intensity. This did not lead to more SAPs being sampled within the plume over the southeastern Atlantic Ocean. In 2018, the BB season started to diminish, as is evident from the reduced frequency of active fires and the southward shift of the fires from the DRC and northern Angola. Nevertheless, more SAPs were observed. Due to differences in the locations of research flights between 2017 and 2018, it is possible that the smaller number of observed SAPs sampled in 2017 was related to sampling statistics rather than differences in SAP concentrations in the plumes.

## 5 Conclusions

This study examined characteristics of supermicron-sized aerosol observed using in situ instruments on board the NASA P-3 Research Aircraft over the southern Atlantic Ocean during the 2017 and 2018 NASA ORACLES field campaigns. Trajectory analyses, using the NOAA HYSPLIT model, heightened concentrations of organics, nitrates, ammonium, and sulfates, a young plume age of 2–3 d, the presence of rBC, and TEM–EDX-identified carbonaceous particles with enhanced K and Si peaks all support the hypothesis that the SAPs were associated with biomass burning within southern central Africa. Similar particles emitted from biomass burning have been observed previously in localized field studies (Chakrabarty et al., 2014) and laboratory experiments (Kearney and Pierce, 2012) but not as far from the source as observed here. This work shows that SAPs can be transported hundreds of kilometers from their source region. This analysis of SAPs focused on the shape, size, and composition of these particles, based on optical array probe imagery, and analysis of aerosol filters. This study examined 15 research flights that recorded SAPs (out of 25) from 2017 and 2018. No SAPs were detected during the 2016 IOPs.

In 2017, 165 SAPs sized 10 to 1520 μm were observed 820 km off the coast of Angola. The 2018 field campaign resulted in 692 SAPs 10 to 1100 μm observed 370–820 km off the coast of Angola. Filters collected during the 2017 campaign, containing particles sampled within the aerosol plume where the SAPs were observed, showed that collocated smaller particles were composed of BC and organics of biomass origin. NOAA HYSPLIT backward trajectories placed the source region of SAPs observed during 12 out of the 15 research flights in Angola for both 2017 and 2018. The SAPs composition was not measured. However, given the source location, the presence of rBC, and the TEM-identified carbonaceous particles, it is hypothesized that, based on observed particle shapes, the SAPs imaged by the 2D-S are examples of supermicron-sized BC aerosol, ash, or unburned plant material. The exact impact of this class of large aerosols on aerosol–cloud interactions or cloud radiative processes is unknown but may be significant because of their apparent residence time in the atmosphere and their surface area. Future sampling of BB aerosol plumes hopefully will provide a better understanding of the life cycle of SAPs and their potential role in radiative processes.

## Figures and Tables

**Table 1.** The 2017 research flights were conducted between 12 and 31 August 2017. All concentrations and temperature values are 5 min averages. Some data values were not available and were left blank. Soot number and mass concentrations were obtained from the single particle soot photometer (SP2). Organics, nitrates, sulfates, and ammonium were obtained from the aerosol mass spectrometer (AMS). Plume age was determined from Hybrid Single-Particle Lagrangian Integrated Trajectory (HYSPLIT) and Weather Research and Forecasting (WRF) models. Note: DRC is the Democratic Republic of Congo.

Date	Flight	Latitude	Longitude	Altitude (m)	Temperature (°C)	No. of SAPs	HYSPLIT source region	Plume age (days)
12 Aug 2017	17RF01	8.5° S	5.0° E	1230	22.4	15	Northern Angola	3
13 Aug 2017	17RF02	9.0° S	4.0° E	2700	12.5	0	Far eastern Angola	2.5
15 Aug 2017	17RF03	8.9° S	5.0° E	2100	20.0	55	Northern Angola	2
17 Aug 2017	17RF04	8.0° S	6.0° W	1800	16.2	0	Gabon	5
18 Aug 2017	17RF05	8.0° S	14.0° W	1800	13.3	0	South America	8
21 Aug 2017	17RF06	8.0° S	3.8° E	2900	16.8	9	Central Angola	2
24 Aug 2017	17RF08	8.0° S	5.0° E	2100	17.5	3	Northern Angola	2
26 Aug 2017	17RF09	5.0° S	5.0° E	4000	13.2	0	Central DRC	3
28 Aug 2017	17RF10	9.5° S	5.0° E	4000	8.0	0	DRC	3
30 Aug 2017	17RF11	9.0° S	5.0° E	3500	9.8	71	Northern Angola	3
31 Aug 2017	17RF12	5.0° S	2.2° W	2500	15.4	12	Central Angola	2

Date	Flight	Organics concentration (μgm^−3^)	Nitrate concentration (μgm^−3^)	Sulfate concentration (μgm^−3^)	Ammonium concentration (μgm^−3^)	Soot number concentration (no. of cm^−3^)	Soot mass concentration (μgm^−3^)
12 Aug 2017	17RF01	–	–	–	–	780	2.10
13 Aug 2017	17RF02	12.0	3.1	4.2	1.8	650	1.75
15 Aug 2017	17RF03	38.0	4.1	5.7	2.1	480	1.10
17 Aug 2017	17RF04	17.0	1.3	2.3	0.6	400	1.20
18 Aug 2017	17RF05	2.1	0.3	2.3	0.6	175	0.41
21 Aug 2017	17RF06	50.0	7.1	12.0	4.5	1250	4.20
24 Aug 2017	17RF08	18.0	2.1	4.2	1.5	1100	3.52
26 Aug 2017	17RF09	25.0	2.0	4.0	1.0	900	2.30
28 Aug 2017	17RF10	–	–	–	–	1600	6.10
30 Aug 2017	17RF11	30.0	4.5	4.0	4.5	1300	5.80
31 Aug 2017	17RF12	50.0	9.0	9.0	5.0	1300	5.00

**Table 2.** The 2018 research flights conducted between 27 September and 23 October 2018. All concentrations and temperature values are 5 min averages. Some data values were not available and were left blank. Soot number and mass concentrations were obtained from the SP2. Organics, nitrates, sulfates, and ammonium were obtained from the AMS. Plume age was determined from HYSPLIT and WRF models. Note: DRC is the Democratic Republic of Congo.

Date	Flight	Latitude	Longitude	Altitude (m)	Temperature (°C)	No. of SAPs	HYSPLIT source region	Plume age (days)
27 Sep 2018	18RF01	11.3° S	5.0° E	1500	21.3	205	Southern DRC	4.5
30 Sep 2018	18RF02	7.9° S	5.0° E	1500	20.2	98	Southern DRC	5
2 Oct 2018	18RF03	7.8° S	5.5° E	2500	15.0	102	Eastern Angola	2
3 Oct 2018	18RF04	7.0° S	6.3° E	1300	19.4	48	Northern Angola	2
5 Oct 2018	18RF05	9.8° S	6.2° E	2100	16.7	39	Central Angola	2
7 Oct 2018	18RF06	10.0° S	5.0° E	2400	16.2	0	Zambia	4
10 Oct 2018	18RF07	13.0° S	5.0° E	2100	21.0	51	Northern Namibia	3
12 Oct 2018	18RF08	2.0° S	6.6° E	2000	17.1	0	Northern Angola	4
15 Oct 2018	18RF09	10.0° S	5.0° E	1600	22.5	98	Southern Angola	3
17 Oct 2018	18RF10	7.4° S	10.5° E	1900	20.0	16	South America	6
19 Oct 2018	18RF11	9.0° S	7.0° E	2500	16.2	35	Southern Angola	3
21 Oct 2018	18RF12	9.0° S	5.0° E	2100	17.3	0	Tanzania	5
23 Oct 2018	18RF13	1.0° S	5.0° E	2000	17.0	0	DRC	3

Date	Flight	Organics concentration (μgm^−3^)	Nitrate concentration (μgm^−3^)	Sulfate concentration (μgm^−3^)	Ammonium concentration (μgm^−3^)	Soot number concentration (no. of cm^−3^)	Soot mass concentration (μgm^−3^)
27 Sep 2018	18RF01	9.0	1.20	3.3	1.30	670	2.70
30 Sep 2018	18RF02	–	–	–	–	452	1.63
2 Oct 2018	18RF03	8.1	0.50	1.2	0.80	350	1.10
3 Oct 2018	18RF04	1.2	0.10	0.8	0.12	200	0.55
5 Oct 2018	18RF05	7.8	0.50	1.6	0.50	475	16.00
7 Oct 2018	18RF06	5.8	0.40	1.2	0.40	420	1.25
10 Oct 2018	18RF07	4.8	0.60	0.9	0.30	355	1.20
12 Oct 2018	18RF08	2.3	0.20	0.3	0.10	94	0.12
15 Oct 2018	18RF09	4.0	0.25	1.1	0.25	475	1.10
17 Oct 2018	18RF10	20.0	3.10	3.0	2.10	1100	2.50
19 Oct 2018	18RF11	3.7	0.20	0.6	0.10	275	0.52
21 Oct 2018	18RF12	–	–	–	–	175	0.55
23 Oct 2018	18RF13	–	–	–	–	176	0.59

## Data Availability

All ORACLES 2017 and 2018 in situ data used in this study are publicly available at https://doi.org/10.5067/Suborbital/ORACLES/P3/2017_V2 (ORACLES Science Team, 2020a) and https://doi.org/10.5067/Suborbital/ORACLES/P3/2018_V2 (ORACLES Science Team, 2020b). The ERA5 data (https://apps.ecmwf.int/data-catalogues/era5/?class=ea; Hersbach et al., 2020) are downloadable.
